# Ectopic cervical thymoma in myasthenia gravis: a case report

**DOI:** 10.1186/s12883-024-03656-6

**Published:** 2024-05-23

**Authors:** Shima Zargar, Maryam Hosseini Farahabadi, Samuel J. Reynolds

**Affiliations:** 1 Department of Neurology, The University of Texas Health Sciences Center at San Antonio, San Antonio, TX USA; 2 Department of Neurology, University of New Mexico Hospital, Albuquerque, NM USA; 3 Department of Pathology, University of New Mexico Hospital, Albuquerque, NM USA

**Keywords:** Myasthenia gravis, Acetylcholine receptor-antibody, Mediastinal mass, Ectopic thymoma

## Abstract

**Background:**

Ectopic cervical thymoma (ECT) is an extremely rare tumor, especially in association with myasthenia gravis (MG).

**Case presentation:**

We report a case of myasthenia gravis with an ectopic thymoma in the neck, whose myasthenic symptoms significantly improved after complete removal of the mass. A 55-year-old woman with generalized myasthenia gravis (MG) experienced worsening neuromuscular weakness after abruptly discontinuing pyridostigmine. Testing revealed acetylcholine receptor-antibody (AChR-Ab) positivity and a cervical mass initially thought to be thyroid or parathyroid was identified as a thymoma, type A. Post-surgery and radiation therapy, her myasthenic symptoms improved significantly with less prednisone and pyridostigmine requirements over time and no need for additional immunotherapies.

**Conclusions:**

Diagnosing ECTs is challenging due to rarity, atypical locations, and inconclusive fine needle aspiration cytology (FNAC) results, often misinterpreted as thyroid or parathyroid lesions. As proper management of patients with MG, including thymectomy, offers favorable clinical outcomes such as significant improvement in myasthenic complaints and reduced immunosuppressive medication requirements, clinicians should be vigilant of the ectopic locations of thymomas to ensure timely diagnosis and intervention.

## Background

Ectopic cervical thymoma (ECT) is an extremely rare tumor. We report a case of myasthenia gravis with an ectopic thymoma in the neck, whose myasthenic symptoms significantly improved after complete removal of the mass. To the best of our knowledge, there have been few similar cases reported in the literature. Therefore, our purpose is to outline the challenges and the importance of diagnosing ectopic thymomas.

## Case presentation

**Fig. 1:**
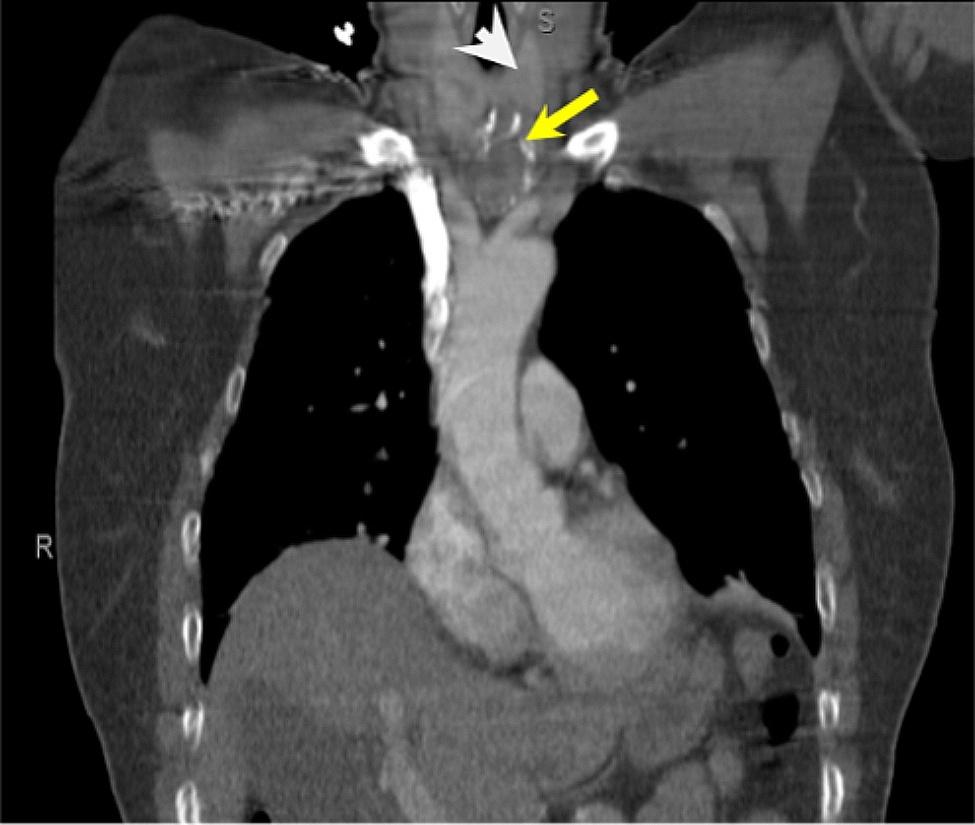
Chest CT-scan shows a heterogenous mass (yellow arrow) with peripheral calcifications measuring 40 × 33 × 28 mm, inferior to the left thyroid lobe (white arrowhead) and extending into the upper mediastinum

A 55-year-old woman with a recent diagnosis of myasthenia gravis (MG) was admitted for worsening neuromuscular weakness after abruptly self-discontinuing pyridostigmine. Review of her records was notable for a two-month history of generalized weakness. A limited workup had been done with a computed tomography (CT) scan of the chest showing a heterogenous mediastinal mass and pyridostigmine was initiated by her primary care physician. Her presenting symptoms and physical examination showed bilateral facial weakness, ptosis, dysconjugate eye movements, neck flexor weakness, dysphagia, shortness of breath, difficulty clearing secretions and bilateral upper and lower extremity weakness. She was admitted to our neuroscience intensive care unit for myasthenic crisis. She required non-invasive ventilation and was treated with intravenous immunoglobulin and steroids. During further workup, the patient tested positive for acetylcholine receptor-antibody (AChR-Ab) and tested negative for muscle-specific kinase (MuSK) antibodies. A contrast-enhanced chest CT-scan found a heterogenous mass with coarse peripheral calcifications measuring 40 × 33 × 28 mm, arising from or immediately adjacent to the inferior left thyroid lobe extending into the upper mediastinum with a resultant right-sided deviation of the trachea (Fig. 1). It was initially confused for a mass of thyroid or parathyroid origins based on its’ location and misleading fine needle aspiration cytology (FNAC) results. FNA cytology showed follicular cells, macrophages, scattered lymphocytes and neutrophils, and calcifications. The cell block was negative for thyroglobulin and TTF-1; therefore, results were in favor of a parathyroid lesion rather than a thyroid mass. However, parathyroid hormone and thyroid-stimulating hormone levels were normal.

**Fig. 2:**
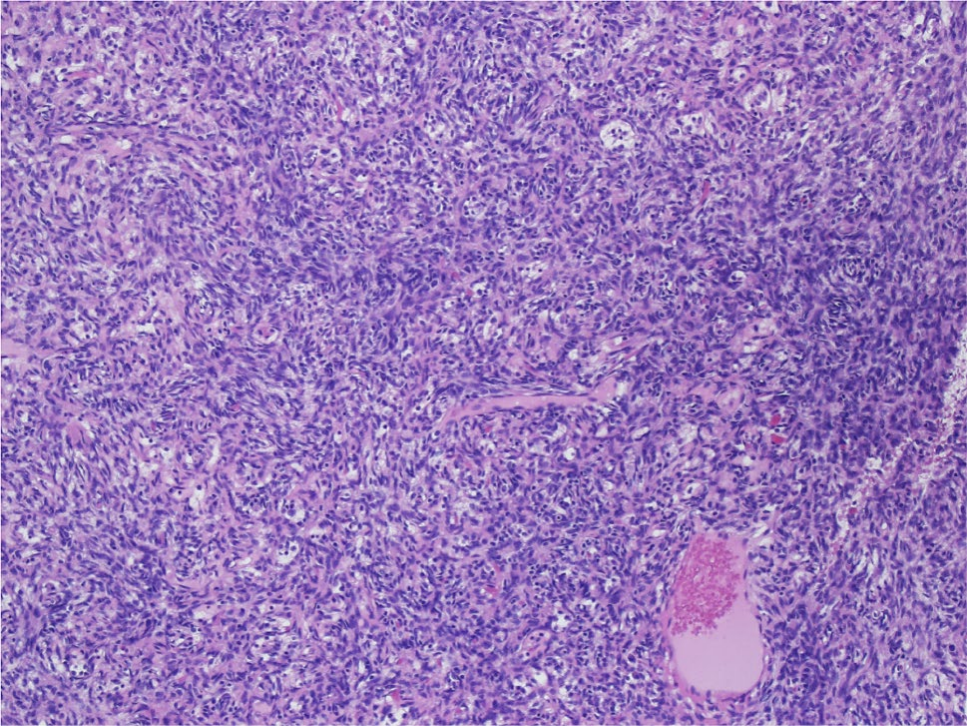
Pathology demonstrating neoplastic proliferation of thymic epithelial cells categorized as a Type A thymoma based on WHO classification

**Fig. 3:**
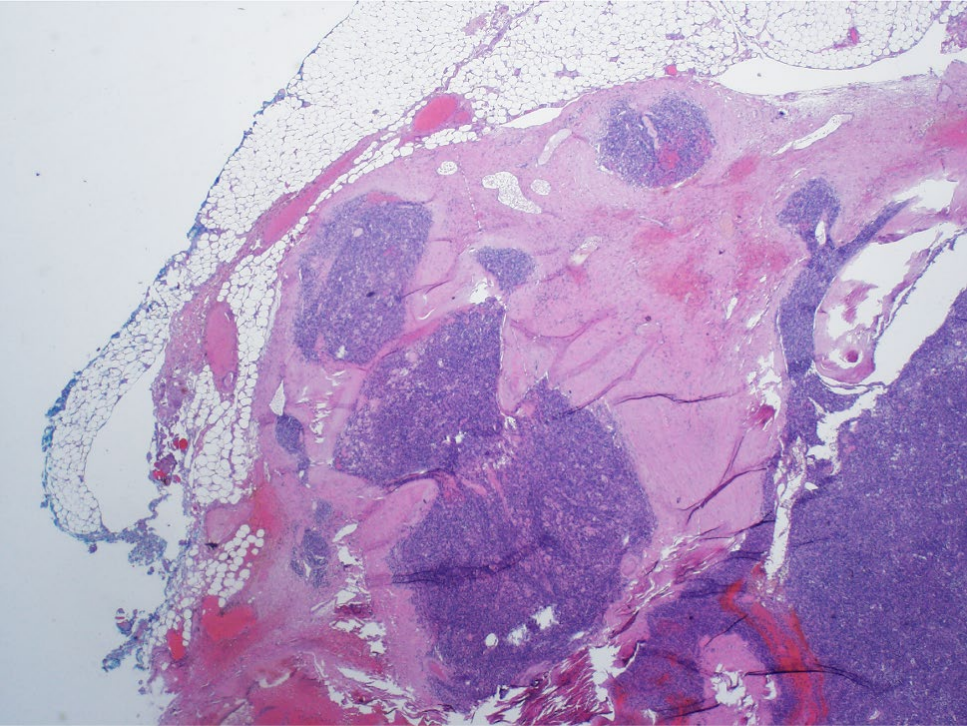
Areas of trans-capsular invasion consistent with a Masaoka stage II thymoma or T1a based on TNM staging

The mass was trans-cervically resected during the same hospitalization. The excised specimen demonstrated a nodular encapsulated piece of tan to dark red-brown soft tissue with both solid and cystic components, and focally calcified. Microscopic examination revealed pancytokeratin and CK5/p63 positive oval-shaped tumor cells consistent with a type A thymoma based on the World Health Organization (WHO) classification (Fig. 2). The presence of microscopic trans-capsular invasion classified the lesion as a Masaoka stage II thymoma corresponding to stage T1a based on the TNM staging system (Fig. 3). The patient was discharged on prednisone 30 mg daily, pyridostigmine 60 mg four times a day, and methotrexate 10 mg per week with a plan to gradually increase to 20 mg per week.

After 3 months, she showed a remarkable improvement in myasthenic symptoms with residual ocular symptoms and mild dysphagia to solid foods that required diet modification to small bites with no weakness noted on strength testing of the arms and legs. Prednisone was decreased to 25 mg daily and pyridostigmine was continued at the same dose. A repeat CT-scan revealed no evidence of recurrence or metastatic disease. Methotrexate was discontinued in anticipation of radiation therapy and the patient subsequently underwent five weeks of intensity-modulated radiation therapy to the postoperative site to prevent recurrence. At 6 months, the patient showed significant improvement in myasthenic symptoms, with only very mild residual dysphagia; therefore, prednisone was further decreased to 10 mg daily and pyridostigmine to 45 mg BID. At 18 months, she was completely taken off prednisone. No additional immunotherapies were required, and no further hospitalizations occurred during the 18-month follow-up period.

## Discussion and conclusions

Thymomas are rare tumors derived from thymic epithelial cells, constituting less than 1% of all adult cancers [1]. These tumors typically develop in the anterior mediastinum [2], however, various aberrant locations have been reported in the literature, such as the neck, carotid triangle [3], chest wall, pleura, lung, and heart [1].

Ectopic thymomas, which arise from remnants of thymic tissue along its embryonic descent pathway, constitute 4% of all thymomas [1, 4]. In 2019, a review of 114 cases of ectopic thymoma reported in the literature, revealed a female predominance (68 out of 114). Nine of these cases were associated with myasthenia gravis, with four of which were in the neck [4].

Ectopic cervical thymomas should be considered in all MG patients with a cervical mass or those with persistent myasthenic symptoms who have undergone a mediastinal thymectomy [5]. In the neck, these tumors are commonly located in the anterior neck or situated below/within the inferior aspect of the thyroid gland, often causing confusion due to their resemblance to thyroid nodules and parathyroid masses [1, 5]. They are most commonly asymptomatic but can rarely present with myasthenia gravis, superior vana cava syndrome, stridor, dyspnea, and/or dysphagia due to tracheal and/or esophageal compression [6]. Misleading fine needle aspiration cytology (FNAC) results can also lead to misdiagnosis [4], which in our case, were initially in favor of a parathyroid lesion. Table 1 offers a concise summary of select cases wherein the uncommon correlation between ectopic cervical thymomas and myasthenia gravis was observed concurrently.

**Table 1:** Ectopic cervical thymomas and myasthenia gravis

Author	Age	Gender	Location of Thymoma	Mediastinal Mass	Clinical manifestations	Fine Needle Aspiration Cytology	Preoperative Diagnosis	WHO Classification post-surgery	Long-term MG outcomes
Sato et al. [7]	74	F	Neck, adjacent to caudal thyroid	Absent	Ease of fatiguability and blepharoptosis	N/A	Thyroid Tumor	Thymoma	Favorable
Choi et al. [8]	53	F	Neck, left thyroid	Absent	Right ptosis and weakness in both upper extremities	Lymphocyte infiltration without thyroid cells	Lymphoma	Type B1 Thymoma	Favorable
Wu et al. [9]	58	F	Neck, below the left thyroid lobe	Present	Ptosis	N/A	Ectopic Thymoma	Type AB Thymoma	Favorable
Kumazawa et al. [10]	47	F	Neck, posterior to right thyroid lobe	Absent	Ptosis, diplopia, fatigable mandibular weakness	Many CD1a positive immature lymphocytes	Ectopic Thymoma	Type B1 Thymoma	Favorable
Marouf et al. [1]	31	F	Neck, lower pole of left thyroid gland	Present	Ptosis, weakness, and rapid fatigue	Mature lymphocytes and epithelial cells	Ectopic Thymoma	Type AB Thymoma	Favorable
Sekiguchi et al. [11]	78	F	Neck	Absent	Dyspnea and shortness of breath	N/A	Ectopic Thymoma	Type B Thymoma	Favorable
Kamimura et al. [12]	61	F	Neck	Absent	Fatigue, ptosis, and dysphagia	N/A	Parathyroid Tumor	Type B2 Thymoma	Favorable

The relationship between thymoma and MG is due to the production of T-cells in the tumor that generate anti-acetylcholine receptor antibodies, thereby contributing to MG. According to current guidelines, thymectomy is strongly recommended for thymomatous MG and non-thymomatous, generalized MG with positive AchR-Ab, to enhance long-term clinical outcomes [13]. Therefore, surgical removal of ectopic thymomas might also hold prognostic value in determining the long-term outcomes of MG [8]. During long-term follow-up, MG patients who underwent surgical removal of their ectopic thymoma experienced complete or pharmacological remission, significant improvement in myasthenic complaints, and reduction of immunosuppressive requirements [1]. Most cases of ECTs have a benign course, rarely developing into a malignant form [5, 8].

In conclusion, thorough medical evaluation in MG patients is crucial, to consider the potential presence of anterior mediastinal thymoma and/or ectopic thymoma. Ectopic thymomas pose diagnostic challenges due to unusual locations, rarity, atypical clinical manifestations, and inconclusive FNAC results, which can lead to misdiagnosis [4]. Similar to thymomas, ectopic thymomas also carry malignant potential.

The significant clinical improvement and reduced medical requirements observed in our patient likely resulted from the early detection and removal of the ectopic thymoma. Thus, early detection and surgical resection, are strongly recommended for favorable long-term outcomes.

## Data Availability

All data is provided within the manuscript.

## Abbreviations

ECT: Ectopic cervical thymoma
MG: Myasthenia gravis
AChR-Ab: Acetylcholine receptor-antibody
CT: Computed tomography
FNAC: Fine needle aspiration cytology
WHO: World Health Organization
TNM: Tumor, Node, Metastasis
ICU: Intensive care unit
